# Acute Psoitis*Read before the Buffalo Medical Club.

**Published:** 1881-12

**Authors:** Herman Mynter


### ACUTE PSOITIS.*

By Herman Mynter, M. D.

Acute inflammation of the psoas muscle is a rare affection, and the literature of the subject is very scarce—so scarce that a great many writers ignore its existence. With the exception of a few short articles in "Ziemssen's Encyclopædia," "Bilroth and Pitha's Surgery," "Smidt's Jahresbericht and Half-yearly Compendium of Medical Science," I have been unable to find any notice of this disease, even in our largest surgical works. I myself have had the opportunity of seeing and treating two such cases, and I therefore take the liberty of presenting the subject for your deliberation, leaving out of consideration the common form of psoas abscess, originating in caries of the spine or pelvis, and taking a chronic course.

Etiology.—The inflammation, it is stated, may be caused by embolism and metastasis, may extend from the neighboring soft

*Read before the Buffalo Medical Club.

parts, from inflammatory processes in the pelvic viscera or the areolar tissue around them.* I doubt whether this statement is wholly correct. The ileo-psoas muscle is so well protected by the strong iliac fascia, that inflammation, originating in the pelvic viscera, will not be apt to take this course, especially as the great amount of loose connective tissue between the pelvic viscera and the peritoneal covering will favor the extension of the inflammation in other directions. More rarely, as I intend to consider in this paper, acute psoitis occurs as an independent disease as the result of violent exertion, straining (during parturition), or direct contusion. Finally it may occur "without any discoverable cause, and is then viewed as rheumatic or due to chill."† The disease is found relatively often in childhood.‡ During 18 years, 29 cases were observed in Vienna, in Franz Joseph's Hospital for Children, among 14,000 cases. The primary acute form is the result in children exclusively of injuries; more boys than girls are affected, and older children oftener than younger ones, as they are more exposed to injuries. The disease is generally only on one side, exceptionally on both, and is often first discovered by post-mortem examination.

Dr. Neureutter does not give any statistics about the number of recoveries of these 29 cases, nor about the pathological condition at the post-mortem examinations, but he mentions one case in a boy, 11 years of age, who as the result of a fall developed a traumatic psoitis as a complication in an interstitial hepatitis. The patient died, and at the post-mortem the abscess was demonstrated in the psoas muscle. Another case, with report of the post-mortem, is reported in *Jahrbuecher fuer Kinderheilkunde*, by Dr. Wittman.§ It occurred at the hospital for children, at Pesth, and as proof of its extreme rarity the doctor mentions that he in the literature can find only three similar cases, reported by Bokai in *Oestreichische Zeitschrift fuer Kinderheilkunde.* Wittman's case

* Ziemssen's Encyclopædia, vol. xvi, p. 96.

† Ibidem.

‡ Dr. Th. Neureutter in Smidt's Jahrbücher, 1878.

§ Smidt's Jahrbücher, 1875.

was that of a boy, 8 years of age, who had injured himself by a mistep during gymnastic exercises. On examination he found high fever, insomnolence, somnolence, great pain in right lumbar and iliac region, and especially at the insertion of the psoas muscle at the thigh. The leg was fixed and adducted (?). Swelling and redness occurred later in the inguinal region, but without fluctuation.

The patient succumbed to a pneumonia, and at the post-mortem the psoas muscle was found infiltrated with fluid, yellowish green matter, as was the connective tissue which covered the psoas and iliacus muscle. Some small pus cavities were found in the adductor muscles.

Of Bokai's three cases, one died and two recovered—one after the formation of an abscess on the thigh, the other almost without suppuration.

That inflammation can occur in muscular tissue is recognized, and especially as the result of some strain or partial rupture. Bryant states that it is not seldom seen in the rectus abdominis as well as in the psoas muscle as a result of injury; "Indeed, as a cause of psoas abscess, I believe injury to be more common than spinal disease." Neither is it to be wondered at that this affection is found especially in the psoas muscle, when we consider that by its size and function this muscle is particularly exposed to strains and injuries, more so than any other muscle in the body.

Symptoms.—The disease commences with fever and severe pain in the lumbar region, sometimes after a period of some weeks after the injury, during which time there have been only indistinct symptoms. The pain soon extends downwards to the thigh. Fever and pain may last for weeks and the diagnosis be doubtful, till the peculiar position of the corresponding leg clears up the doubt. Since active contraction of the inflamed muscle is as painful as passive extension, the patient relaxes it as much as possible, and consequently flexes the thigh and rotates it outwards.

We often find spasmodic rigidity of the affected muscles, due to reflex irritation of sensory nerve ends, or to direct irritation of the motor fibres of the muscular tissue itself.* When the abnormal position is maintained for some time, shortening of the muscles may take place. Pressure in the iliac region along the psoas muscle is extremely painful, as is pressure in the lumbar region. By deep palpation we will, generally, in course of time, be able to feel an obscure swelling of oblong form, and sometimes we may detect fluctuation.

The exudation takes place either in the muscle itself, which then may be perfectly destroyed, and parts of muscular tissue be found mixed with the matter, or on the surface of the muscle beneath the iliac fascia. Pitha says that in this case a fluctuating abscess will quickly appear in the abdominal wall above Poupart's ligament. At last the matter rushes down on the thigh, and, according to Shaw, invariably on one place, behind Poupart's ligament, and between the united tendons of the iliacus internus and the psoas muscle and anterior inferior spinous process of the ilium. The situation corresponds with the junction of the outer with the middle third of Poupart's ligament.† As the abscess escapes from the abdomen, it becomes released from compression and enlarges in Scarpa's triangle. It then, if left to itself, generally passes downwards and inwards along the sartorius muscle. Sometimes it turns inwards over the adductor muscles, rarely outwards. Occasionally it divides in the groin, one portion going inwardly, another outwardly.

The opening between the abdominal and femoral portion is necessarily always on the outer side of the femoral vessels, but once arrived on the thigh, the abscess occupies in different cases different relations to the femoral vessels. In one case it may be in front, in another on the outer side, on the inner side, on both sides and in front, behind and lifting up the femoral vessels, so that their pulsation may be felt immediately beneath the super-

* Ziemssens' Encycl., vol. xvi., p. 97.

† Halfyearly Compendium of Medical Science, January, 1875.

ficial covering and simulate an aneurism, as it occurred to Dupuytren.

Occasionally the abscess may, as is stated in Ziemssen, take another course and open above the crist of the ilium, into the pleura, pass out through incisura ischiatica, or forward between fascia and musculus transversalis, open into the rectum, vagina, etc., etc., but the regular course is the one described. The fever is, according to Ziemssen, always present from the outset, is not of any regular type except when matter has formed, when it takes the type of suppurative and pyæmic fever. The pain in the muscle is often associated with pains in the course of the nerves, which perforate the psoas, ileo-hypogastrica, ileo-inguinal, genito-crural and external cutaneous. The abscess may remain circumscribed and finally lead to cicatrization, or the pus may become inspissated and enclosed in a capsule, but it will be very exceptionally.

The prognosis is doubtful, but at any rate more favorable than the chronic form of psoas abscess. If of metastatic nature, the result is always death, but if the result of injury, or of rheumatic nature, and discovered in time, the prognosis must be considered favorable. In regard to diagnosis, little need to be said. The acute attack with continuous fever, the local tenderness in the lumbar and iliac region, the obscure swelling, corresponding to the psoas muscle, the characteristic position of the limb, the symptoms on the part of the nerves, ought to insure a correct diagnosis after some time has passed. Inflammation of the hip-joint may be excluded by the lack of pain and change of form of the posterior part of the hip, and by the previous history. Aneurism and femoral hernia are mentioned in larger surgical works in regard to the diagnosis of the chronic psoas abscess. I doubt if these could ever be confounded with an acute abscess. Inflammations of the pelvic viscera may be excluded by vaginal examination and by their more superficial position. In one of my cases a swelling was felt over the rim of the pelvis, corresponding to the median margin of the psoas. The treatment is purely surgical, and as soon as possible the operation ought to be performed. Till that point arrives, the disease should be treated on general surgical principles.

This operation consists in introducing an aspirator needle into the psoas muscle, commencing in Scarpa's triangle below Poupart's ligament, and then, bringing the needle in a horizontal position, letting it follow the course of the muscle upward, inward and backward, till the matter is found. A free incision must then be made with the needle as a guide, drainage tube introduced, and the cavity treated in the usual way. Allow me, finally, to report two cases I have seen during the last year.

*Case 1.* Mrs. D., 54 years of age, a healthy German widow, who lived under very favorable circumstances, was seen by Dr. Shade, on April 12th, 1880. Six weeks previously she had an attack of acute gastritis with vomiting, diarrhœa and fever, from which she recovered perfectly. She complained then, without any known cause, of deep-seated pain of neuralgic character in the left lumbar region, extending downwards to the thigh. Pulse, 104; temperature, 101½.

By objective examination nothing particular was discovered, except that deep palpation in left lumbar region was very painful. A mixture of aconite and morphia in a saturated solution of citrate of potash was ordered, and a hot poultice applied, and the symptoms became thereafter less prominent, while a moderate fever continued.

April 26th. A slight swelling was noticed in left lumbar region by deep palpation, and very painful for pressure.

May 1st. The swelling and deep-seated pain extend downward and are most noticeable in the iliac region. Her leg was kept flexed in the hip-joint and rotated outward. Extension was impossible on account of pain. The patient was sleepless and restless, no appetite, continuous fever (about 102), increasing weakness, feeling of numbness of left femur. These symptoms continued and gradually increased, while the strength decreased in spite of quinine, tonics and appropriate nourishment.

May 6th. I saw the patient in consultation with Dr. Shade. She was very emaciated, temperature 102, pulse 108. .Left leg extremely flexed and rotated outwards, and could not be extended, while slight flexion was possible without pain. Slight movements of the hip-joint and pressure on trochanter major, the knee and posterior part of the joint not painful. In left iliac region a deep-seated swelling was felt, very painful on pressure; fluctuation could not be discovered. Nothing abnormal in Scarpa's triangle. By vaginal examination the uterus and its appendages were felt in a normal state. The case was considered an acute psoas abscess, and the treatment continued for a few days to let the symptoms develop.

May 8th. A diffuse fluctuating swelling appeared below Poupart's ligament outside the femoral vessels. Under chloroform, Drs. Shade and Granger being present, an incision was made and about ½ pint of good and laudable pus evacuated. The finger could then be introduced high up behind Poupart's ligament and made to explore a large pus cavity in the psoas muscle. The inner opening was enlarged, a drainage tube introduced, the cavity syringed out with carbolized water, and antiseptic dressing applied. The farther course was favorable. The fever disappeared, the appetite returned, and in the course of 14 days the drainage tube was removed, and in a week more the wound was healed. The contraction of the muscles gradually disappeared, and the patient has since been perfectly well.

*Case 2.* Mrs. T.; 28 years of age; was confined with her fifth child December 1st, 1880. The delivery was easy, a midwife being in attendance. Four days after her confinement there was slight pain in the right lumbar region, which grew less in a few days without any treatment, but soon returned again. Sixteen days after her confinement, Dr. Hebenstreit was called in, and found the patient sick in bed, complaining of deep-seated pain in right lumbar and iliac region, with more or less superficial tenderness. Temp. 102, pulse, 108. Local application of warm aromatic lotions and internal administration of anodynes again made the symptoms disappear in a few days, and the patient was discharged until further notice. Dr. Hebenstreit was called in again January 3d, 1881, and found the former symptoms aggravated, the patient sleepless and restless, continual deep-seated, throbbing pain in the right lumbar and iliac region, with intense tenderness of the abdominal wall. Temp. 103, pulse 108. The right leg slightly contracted and rotated outwards, could not be fully extended. From now till January 22d, when I saw the patient in consultation with Dr. Hebenstreit, the condition slowly but gradually grew worse. Pain was excessive, lancinating downward to the knee, and required large amount of morphia. The muscles contracted more and more, the limb was now bent in an acute angle in the hip-joint and rotated outwards. A deep-seated swelling could be felt in the lumbar region, extending downwards into the iliac region. Fever had been continuous, with morning remissions, ranging between 102 and 104. Fluctuation could not be felt, and the tenderness was so extreme that no perfect examination could be performed without anæsthesia. Examination of vagina showed the uterus and its appendages free from soreness, and in a normal state.

Over the rim of the pelvis, on the right side, a local tenderness, corresponding to the psoas muscle. The diagnosis being considered acute psoas abcess, operation was decided upon and attempted January 24, 1881, in the presence of Drs. Tobie, Dayton, Hauenstein and Hebenstreit. A small-sized but long trocar was introduced vertically below Paupart's ligament, in the outer part of Scarpa's triangle, and having perforated the fascia lata, the trocar was brought in a horizontal position, and carried into the psoas muscle upwards, inwards and backwards behind Paupart's ligament, a distance of about four inches. By removing the needle, healthy matter flowed out, showing the correctness of the diagnosis.

A transverse incision was now made below Paupart's ligament through fascia lata, and with the trocar as guide, a large opening was made and the finger introduced into the pus cavity. About one pint of healthy matter was evacuated. A drainage tube was intioduced 12 inches, the cavity washed out with warm carbolized water, and antiseptic dressing applied. The fever disappeared immediately and the patient gained ground daily.

February 11th. A severe chill occurred to-day with high temperature (105), after the patient, for a couple of days, had been left to the tender care of her mother. By perfectly cleaning the cavity, the fever disappeared in a few days.

February 15th. Drainage tube removed on account of pressure on crural nerve, and a smaller one introduced. The cavity measures still 12 inches.

The patient continued to improve, and was discharged cured three weeks later.

The contraction of the hip-joint gradually disappeared, and the patient has since been well.

